# Epilepsy and Intellectual Disability in a Boy with a 2q13 Microdeletion Affecting the *BUB1* Gene

**DOI:** 10.3390/pediatric18040096

**Published:** 2026-07-12

**Authors:** Verónica Judith Picos-Cárdenas, Roberto Iván Avendaño-Gálvez, Alberto Kousuke De la Herrán-Arita, Loranda Calderón-Zamora, Salvador Cervín-Serrano, José Alfredo Contreras-Gutiérrez, Dora María Cedano-Prieto, Juan Pablo Meza-Espinoza

**Affiliations:** 1 Facultad de Medicina, Universidad Autónoma de Sinaloa, Culiacán 80019, Sinaloa, Mexico; veronicapicos@uas.edu.mx (V.J.P.-C.); alberto.kousuke@uas.edu.mx (A.K.D.l.H.-A.);; 2 Centro de Rehabilitación y Educación Especial (CREE), Sistema DIF Sinaloa, Culiacán 80020, Sinaloa, Mexico; salud.sectec@gmail.com; 3 Facultad de Biología, Universidad Autónoma de Sinaloa, Culiacán 80040, Sinaloa, Mexico; 4 Centro de Investigación en Docencia en Ciencias de la Salud, Universidad Autónoma de Sinaloa, Culiacán 80030, Sinaloa, Mexico; 5 Facultad de Ciencias Químico-Biológicas, Universidad Autónoma de Sinaloa, Culiacán 80010, Sinaloa, Mexico; dora.cedano@uas.edu.mx; 6 Facultad de Medicina Matamoros, Universidad Autónoma de Tamaulipas, Sendero Nacional km 3, Col. San José, Matamoros 87300, Tamaulipas, Mexico

**Keywords:** epilepsy, 2q13 microdeletion, *BUB1* gene, 11q21 microdeletion

## Abstract

**Background**: The chromosomal microdeletion syndrome 2q13 is characterized by craniofacial dysmorphism, developmental delay, intellectual disability, autism spectrum disorder, attention deficit hyperactivity disorder, cardiac abnormalities, and seizures. **Case Presentation**: In this study, we present a descriptive genomic observation of a teenage boy presenting with epilepsy, intellectual disability, and mild facial dysmorphism, found to carry a 48.55 kb 2q13 microdeletion restricted to the *BUB1* locus alongside a concurrent 11q21 microdeletion. While his clinical features overlap with the 2q13 microdeletion spectrum, the exact pathogenic contribution of each variant remains a subject of hypothesis due to the lack of parental inheritance data. **Conclusions**: Further research is necessary to ascertain the impact of the concurrence of small deletions on these disorders. This case underscores the clinical complexity introduced by compound minor copy number variations and emphasizes the value of molecular cytogenetics in evaluating idiopathic neurodevelopmental disorders.

## 1. Introduction

Copy number variations (CNVs) are defined as genomic structural alterations involving segments larger than 1000 base pairs, which frequently encompass multiple protein-coding genes [1]. A subset of CNVs has been associated with neurodevelopmental disorders, including autism spectrum disorder (ASD), attention deficit hyperactivity disorder (ADHD), developmental delay, and schizophrenia. The most frequently reported CNVs are deletions and duplications of the 2q13 chromosomal region [2]. The 2q13del CNV has been associated with microdeletion syndrome, a rare chromosomal alteration that results in the loss of several genes. It is estimated that 0.013% of the general population carries this CNV, which has nearly 17% penetrance [2], and exhibits a wide range of clinical symptoms. Patients may present with developmental delays, cognitive impairments, ASD, ADHD, hypotonia, dysmorphic features, congenital heart defects, seizures, and macrocephaly or microcephaly [3,4,5,6]. While the documented number of affected individuals remains below 100, the typical 2q13 microdeletion ranges from 1.35 to 2.1 megabases (Mb). Most of these deletions are approximately 1.7 Mb in size and overlap a 1.3 Mb region (chr2:111,449,141-112,746,937; GRCh37/Hg19) encompassing the *ACOXL*, *BCL2L11*, *ANAPC1*, and *MERTK* genes [5]. This multigenic landscape typically complicates the pinpointing of distinct causative loci. In this study, we document a descriptive case of a teenage boy presenting with epilepsy, intellectual disability, and mild facial dysmorphism who was found to harbor two independent, novel CNVs of uncertain significance (VUS): a miniature 48.55 kb 2q13 microdeletion restricted to the *BUB1* locus and a concurrent 106.19 kb microdeletion in the 11q21 region.

## 2. Case Presentation

The patient is a 14-year-old boy whose parents are not blood relatives and are both 38 years old. He is the product of a second uneventful pregnancy and has two healthy siblings, aged 19 and six. He was delivered by cesarean section at 38 weeks. At birth, he weighed 2950 g, measured 49 cm, and immediately cried. He presented with psychomotor delay, holding his head up at six months and walking at 18 months. His speech is poor. The clinical onset was at age four, marked by generalized seizures involving muscular rigidity, hypertonia, ocular retrusion, and perioral cyanosis. Since then, he has had seizures about every three to six months, some of which have been fever-related. He was prescribed valproate (25 mg/kg/day), levetiracetam (40 mg/kg/day), lamotrigine (1.0 mg/kg/day), and fluoxetine (10 mg/day). In addition to epilepsy, the patient exhibits intellectual disability and a subtle facial dysmorphism, specifically a rounded facial profile, prominent eyebrows, and malar freckling extending to the temporal regions. He also has dental malposition, including a diastema between his incisors and unerupted canines. The patient also has hyperlaxity in both elbows and superficial veins on the anterior face. Electroencephalography, computed axial tomography, and simple and contrast-enhanced magnetic resonance imaging scans of the brain were performed. All scans revealed normal structural and electrical results (imaging profiles were provided for peer review evaluation).

## 3. Genetic Studies

**Figure 1:**
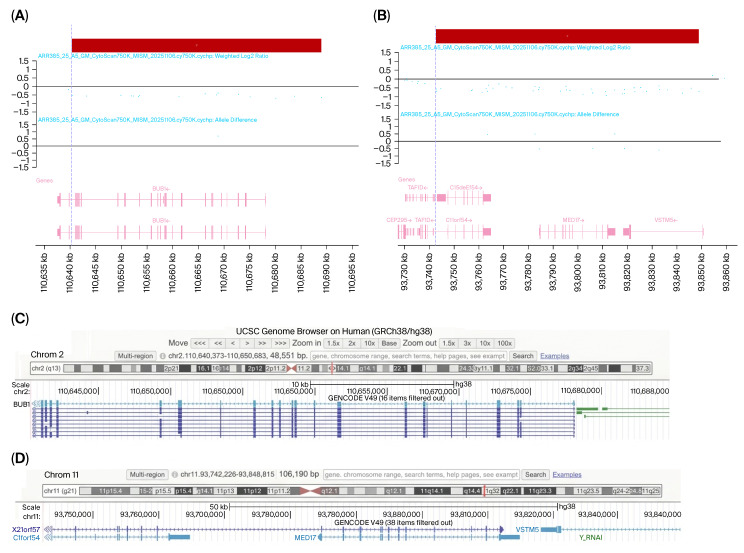
Diagram showing the microdeletions detected in the patient. (**A**,**B**) Plots of the weighted log2 ratio of the microdeletions on chromosomes 2 and 11, respectively. (**C**,**D**) Images obtained from the UCSC Genome Browser in chromosomal build GRCh38/hg38 showing the coordinates of the microdeletions.

GTG-banded karyotyping of the patient’s peripheral blood lymphocytes revealed a normal 46,XY karyotype. Both parents also had normal karyotypes. Array-CGH using CytoScan Technology™ identified a 48.55 kb deletion at 2q13 involving the *BUB1* locus, [GRCh38(Chr2):g.110,640,373-110,688,923)], and an additional 106.19 kb deletion at 11q21 encompassing the *BKGD*, *MED17*, and *VSTM5* genes [GRCh38(Chr11):g.93,742,626-93,848,815] (Figure 1). An analysis of the Invitae^TM^ Epilepsy Panel, which tests 1122 genes, revealed only the heterozygous *MED17* deletion; no additional pathogenic variants were identified. The parents declined to undergo aCGH studies and refused to consent to the publication of photographs of their son.

## 4. Discussion

The 2q13 microdeletion is generally classified as having uncertain significance; however, several cases with a clinical phenotype have been reported, and its penetrance has been estimated to be around 17% [2]. Conversely, the identification of dual microdeletions poses a significant diagnostic challenge, as it is difficult to attribute specific clinical features to a particular region. While the patient’s presentation (facial dysmorphism, intellectual disability, and epilepsy) clinically overlaps with the established 2q13 microdeletion syndrome spectrum [5], the minuscule size of this deletion and the absence of parental data mean its pathogenicity remains entirely unverified. Consequently, this variant must currently be classified as a VUS, and its co-occurrence with the phenotype serves purely as a descriptive observation.

To evaluate the clinical contribution of the concurrent genomic alteration, we analyzed the 106.19 kb 11q21 microdeletion across major databases. This focal variant is not documented in ClinVar, DECIPHER, or gnomAD as an established pathogenic event, meaning it lacks a standardized disease classification and is best interpreted as a VUS. Furthermore, population metrics from gnomAD and dosage constraint scores from ClinGen show no evidence that *BKGD*, *MED17*, or *VSTM5* are haploinsufficiency-sensitive loci. Although larger chromosomal deletions encompassing the 11q21 region are classified in ClinVar as pathogenic (e.g., GRCh38/hg38: 11q13.5-22.1, loss 22.42 Mb; 11q14.1-22.3, loss 28.55 Mb; 11q14.1-22.2, loss 17.68 Mb; 11q14.3-22.3, loss 18.51 Mb), these span hundreds of genes and are distinct from our patient’s case. Nevertheless, three 11q21 genes were lost. Pathogenic variants in *MED17* are linked to infantile cerebral and cerebellar atrophy with severe seizures [7], but this phenotype follows a strict autosomal recessive inheritance model, making clinical symptoms from a single heterozygous deletion unlikely unless paired with an occult mutation on the trans allele, which did not occur in this case. Similarly, *VSTM5* plays a functional role in neuronal morphogenesis, neural circuit migration, and central brain development [8]. Although there is no evidence that these specific genes are sensitive to single-copy loss, a possible synergistic effect arising from the concurrent 11q21 deletion on the neurological phenotype cannot be entirely ruled out. The precise origin of these microdeletions could not be ascertained, as the parents refused to undergo microarray testing. Research indicates that the 2q13 microdeletion is predominantly transmitted from a parent, accounting for approximately 82% of the observed cases [6]. This case represents the shortest known CNV mapping to a 2q13 microdeletion interval to date. However, given that classical *BUB1*-related developmental disorders are strictly autosomal recessive [9], and microcephaly is absent in our patient, there is currently no genetic evidence to support *BUB1* haploinsufficiency as a dominant driver of this phenotype. Rather, this case should be interpreted as a hypothesis-generating observation, where the clinical presentation may instead be governed by unidentified non-coding variants, multi-genic synergistic mechanisms, or unrelated etiologies. On the other hand, due to the role of *BUB1* in mitosis, mutations in this gene are associated with cancer. In this case, however, there was no family history of oncological diseases on either parental side.

Most studies on CNVs in 2q13 show the loss of seven protein-coding genes: *BUB1*, *ACOX1*, *BCL2L11*, *ANAPC1*, *MERTK*, *TMEM87B*, and *FBLN7*, along with other genes at 2q14.1, such as *ZC3H8* and *ZC3H6* [6,10,11,12,13,14,15,16]. However, other studies have reported the deletion of genes beyond *ZC3H6* [3,4,5,17]. Genotype–-phenotype correlations in 2q13 deletions remain elusive, and the marked clinical heterogeneity suggests that phenotypic severity does not strictly correlate with deletion size. It ranges from healthy individuals to those with several clinical manifestations. The main clinical features of this syndrome are craniofacial dysmorphia (80%), developmental delay/intellectual disability (79%), ADHD (48%), hypotonia (44%), ASD (33%), heart defects (31%), macrocephaly (29%), seizures (26%), and microcephaly (23%) [5]. Other recurring abnormalities include glue ear, sleep disturbances, joint pain, joint hypermobility, ear infections, aggressive behavior, gastro-esophageal reflux, chronic constipation, ventriculomegaly, and hydrocephalus [5]. Additionally, a case of oculoauriculovertebral spectrum disorder has been reported [13]. Attempts to explain phenotype in these patients suggest that the *ACOXL* and *BCL2L11* genes may contribute to neurodevelopmental disorders and ASD [11]; the *ANAPC1* and *MERTK* genes have been associated with schizophrenia [18]; and the *FBLN7* and *TMEM87B* genes have been linked with heart defects and craniofacial abnormalities [17]. Although one might hypothesize a potential role for *BUB1* gene involvement based on locus geography, treating this single-copy loss as a confirmed pathogenic driver is highly speculative. Moreover, studies of patients with 2q13 microdeletion syndrome and the typical clinical characteristics have shown that this gene is not consistently affected [4,5,17,19]. In addition, a study [20] reported a 96.65 kb 2q13 microdeletion (Hg18; Chr2:110,219,766-110,316,416) involving the *NPHP1* gene (Hg38; Chr2:110,123,348-110,205,013) in a patient with pontine tegmental cap dysplasia who did not exhibit the typical characteristics of 2q13 microdeletion syndrome [20]. The observed heterogeneity in this and other microdeletion syndromes may be partly explained by the presence of other pathogenic variants, such as additional CNVs or single-nucleotide alterations, as well as the influence of environmental factors [6]. As demonstrated by Yu et al. (2016) [11] and Russell et al. (2014) [17], biallelic deletion of the *TMEM87B* gene exacerbates the phenotype in patients with a 2q13 microdeletion. While it has been hypothesized that the *TMEM87B* and *FBLN7* genes play important roles in heart and craniofacial development [17], we cannot assume that cardiac and craniofacial manifestations result from the loss of these genes, as not all patients who have lost *TMEM87B* and *FBLN7* exhibit these conditions. Some cases of 2q13 microdeletion presenting with additional genomic alterations have been reported (Table 1), but it remains unclear whether these alterations are common and how they influence the phenotype. Nevertheless, the presence of two or more CNVs could exacerbate the clinical manifestations of these disorders. Although the primary cause of the patient’s phenotype remains unknown, this case highlights the need to conduct targeted functional studies on genes associated with 2q13 microdeletions and concurrent CNVs. Such studies will clarify the molecular etiology and variable expressivity of this syndrome.

## 5. Conclusions

In conclusion, this report provides a descriptive genomic characterization of the smallest reported dual-locus microdeletion observation involving the 2q13 and 11q21 regions, with the former restricted entirely to the *BUB1* locus. Because parental segregation and functional validations are absent, both minor CNVs must be clinically designated as VUS. This case does not demonstrate causality for *BUB1* haploinsufficiency, but rather underscores the necessity of molecular tools like aCGH to map out incidental or complex genomic variations in individuals with idiopathic neurodevelopmental conditions.

## 6. Limitations

The main limitation of this case report is that the parents declined to undergo microarray studies. Therefore, we cannot determine if these microdeletions were inherited or de novo, which precludes confirmation of pathogenicity.

## Figures and Tables

**Table 1:** Documented cases of 2q13 microdeletion syndrome and additional genomic variations.

Reference	Present Case	Wolfe et al. [5]	Elron et al. [6]	Yu et al. [10]	Yu et al. [10]	Yu et al. [10]	Yu et al. [10]	Digilio et al. [14]
Sex (Case)	M	M (18)	M (20)	F (1)	F (3)	F (6)	M (8)	NR (4)
Age	14	13	NR	2	6	4	13	NR
del2q13	110,640,373-110,688,923 (Hg38)	111,400,649–113,115,980 (Hg19)	111,392,259–113,143,464 (Hg19)	111,392,197–113,102,594 (Hg19)	111,392,197–113,102,594 (Hg19)	111,142,809–112,762,656 (Hg18)	111,115,715–112,724,494 (Hg18)	111,399,243–113,102,594 (Hg19)
Deletion size	48.55 kb	1.72 Mb	1.75 Mb	1.71 Mb	1.71 Mb	1.62 Mb	1.61 Mb	1.7 Mb
Lost genes	*BUB1*	*BUB1*, *ACOXL*, *BCL2L11*, *ANAPC1*, *MERTK*, *TMEM87B*, *FBLN7*, *ZC3H8*, and *ZC3H6*	*BUB1*, *ACOXL*, *BCL2L11*, *ANAPC1*, *MERTK*, *TMEM87B*, *FBLN7*, *ZC3H8*, *ZC3H6*, and *RGPD8*	*BUB1*, *ACOXL*, *BCL2L11*, *ANAPC1*, *MERTK*, *TMEM87B*, *FBLN7*, *ZC3H8*, *ZC3H6*	*BUB1*, *ACOXL*, *BCL2L11*, *ANAPC1*, *MERTK*, *TMEM87B*, *FBLN7*, *ZC3H8*, *ZC3H6*	*BUB1*, *ACOXL*, *BCL2L11*, *ANAPC1*, *MERTK*, *TMEM87B*, *FBLN7*, *ZC3H8*, *ZC3H6*	*BUB1*, *ACOXL*, *BCL2L11*, *ANAPC1*, *MERTK*, *TMEM87B*, *FBLN7*, *ZC3H8*	*BUB1*, *ACOXL*, *BCL2L11*, *ANAPC1*, *MERTK*, *TMEM87B*, *FBLN7*, *ZC3H8*, *ZC3H6*
Origin	ND	Pat	ND	Mat	Mat	Mat	ND	Mat
AGV	del(11q21)	dup(8)(p23.1)	del(16p11.2)	dup(2p22)	dup(22q11.2)	dup(1q44)	dup(1p36)	dup(6p21.1)
Size	106.19 kb	ND	1.78 Mb	709.36 kb	2.62 Mb	1.19 Mb	4.56 Mb	200.8 kb
AGV origin	ND	ND	ND	Mat	Pat	Pat	Pat	Pat
FD	+	+	+	+	ND	+	ND	ND
HA	-	-	ND	+	-	-	ND	-
Mac	-	-	-	+	+	-	ND	ND
Mic	-	-	+	-	-	+	ND	ND
Seizures	+	-	ND	ND	ND	-	ND	ND
Hypotonia	-	-	ND	-	+	-	ND	ND
DD	-		ND	-	+	+	ND	ND
ADHD	-	+	ND	ND	+	-	ND	ND
ASD	-	+	ND	ND	ND	-	ND	ND
ID	+	-	ND	ND	ND	+	ND	ND
Other features	Diastema between the incisors and hyperlaxity in elbows	Glue ear	ND	Anus displacement	Lacking coordination to jump/run	Hydrocephalus/ large ventricles	ND	ND

Notes. Mat: maternal origin. Pat: paternal origin. ND: not determined (indicates that testing or clinical details for this feature were unavailable or not pursued in the original study, rather than confirmed absent). NR: not reported. GRB: genome reference build. FD: facial dysmorphism. HA: heart anomalies. Mac: macrocephaly. Mic: microcephaly. DD: developmental delays. ADHD: attention deficit hyperactivity disorder. ASD: autism spectrum disorder. ID: intellectual disability. AGV: additional genomic variations: GRCh38(Chr11):g.93,742,626-93,848,815del; GRCh37:16p11.2(28,411,409-30,195,356)x1; GRCh37:2p22(32,480,016-33,189,373)x3; GRCh37:22q11.21(17,270,271-19,891,514)x3; NCBI36:1q44(245,457,485-246,645,245)x3; NCBI 36:1p36(751,796-5,311,663)x3; GRCh37:6p21.1(45,678,713-45,879,492)x3.

## Data Availability

Data is contained within the article.

## Abbreviations

The following abbreviations are used in this manuscript:

aCGH	Array comparative genomic hybridization
ADHD	Attention deficit hyperactivity disorder
AGV	Additional genomic variations
ASD	Autism spectrum disorder
CNV	Copy number variation
DD	Developmental delays
FD	Facial dysmorphism
GRCh37/Hg19	Genome reference consortium/Human genome 19
GRCh38/Hg38	Genome Reference Consortium/Human genome 38
HA	Heart anomalies
ID	Intellectual disability
kb	Kilobases
Mb	Megabases
